# Supplementary material for: Structural bioinformatic study of human mitochondrial respiratory integral membrane megacomplex and its AlphaFold3 predicted water-soluble QTY megacomplex analog
Source: QRB Discov. 2025 Feb 5;6:e12. doi: 10.1017/qrd.2025.2 (PMC11950790; doi:10.1017/qrd.2025.2)

Supplementary Information

Structural bioinformatic study of human mitochondrial respiratory integral membrane megacomplex and its AlphaFold3 predicted water-soluble QTY megacomplex analog

Edward Chen and Shuguang Zhang

Figure S1. The enlarged protein sequence alignments of six integral membrane enzymes with their water-soluble QTY analogs from Figure 1. The symbols | and * indicate whether amino acids are identical or different, respectively. Please note the Q, T, and Y amino acids (red) replacing L, V and I, and F, respectively. The alpha helices (blue) are shown above the protein sequences. The characteristics of natural and QTY analogs listed are isoelectric focusing (pI), molecular weight (MW), total variation %, and transmembrane variation %. Although there are significant QTY changes in the TM alpha helices (26.09–66.67%), their changes in MW and pI are insignificant.

The alignments are: a) NDUA1 vs NDUA1^{QTY}, b) NDUA3 vs NDUA3^{QTY}, c) NDUAB vs NDUAB^{QTY}, d) NDUAD vs NDUAD^{QTY}, e) NDUB1 vs NDUB1^{QTY}, f) NDUB3 vs NDUB3^{QTY}, g) NDUB4 vs NDUB4^{QTY}, h) NDUB5 vs NDUB5^{QTY}, i) NDUB6 vs NDUB6^{QTY}, j) NDUB8 vs NDUB8^{QTY}, k) NDUBB vs NDUBB^{QTY}, l) NDUC1 vs NDUC1^{QTY}, m) NDUC2 vs NDUC2^{QTY}, n) NU1M vs NU1M^{QTY}, o) NU2M vs NU2M^{QTY}, p) NU3M vs NU3M^{QTY}, q) NU4M vs NU4M^{QTY}, r) NU5M vs NU5M^{QTY}, s) NU6M vs NU6M^{QTY}, and t) NU4LM vs NU4LM^{QTY}.

a) NDUA1 vs NDUA1^{QTY}

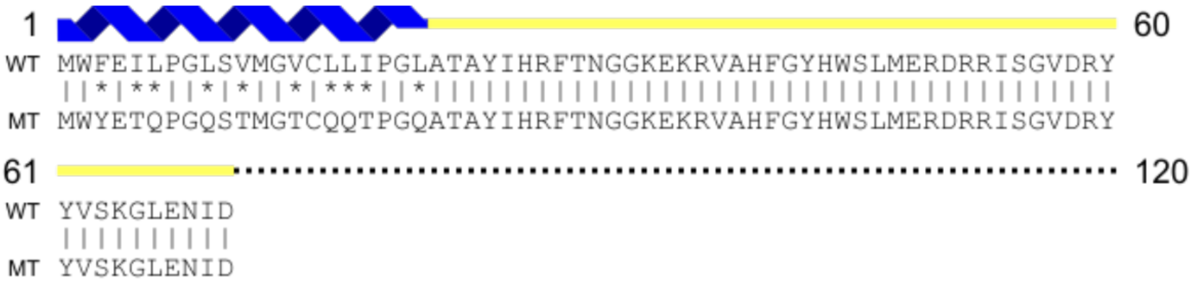

b) NDUA3 vs NDUA3^{QTY}

```
1                                                              60
WT  AARVGAFLKNAWDKEPVLVVSFVVGGLAVILPPLSPYFKYSVMINKATPYNYPVPVRDDG
    |||||||||||||||||***|***||*|***||*|||*||||||||||||||||||||||
MT  AARVGAFLKNAWDKEPVQTTSYTTGGQATTQPPQSPYYKYSVMINKATPYNYPVPVRDDG

61                                                             120
WT  NMPDVPSHPQDPQGPSLEWLKKL
    |||||||||||||||||||||||
MT  NMPDVPSHPQDPQGPSLEWLKKL
```

c) NDUAB vs NDUAB^{QTY}

```
1                                                              60
WT  APKVFRQYWDIPDGTDCHRKAYSTTSIASVAGLTAAAYRVTLNPPGTFLEGVAKVGQYTF
    ||||||||||||||||||||||||||*||*||*||||||*|*|||||||||||||||||*
MT  APKVFRQYWDIPDGTDCHRKAYSTTSTASTAGQTAAAYRTTQNPPGTFLEGVAKVGQYTY

61                                                             120
WT  TAAAVGAVFGLTTCISAHVREKPDDPLNYFLGGCAGGLTLGARTHNYGIGAAACVYFGIA
    ||||*||**|*|||*|||*|||||||||||||||||||||||||||||||||||||||||
MT  TAAATGATYGQTTCTSAHTREKPDDPLNYFLGGCAGGLTLGARTHNYGIGAAACVYFGIA

121                                                            180
WT  ASLVKMGRLEGWEVFAKPKV
    ||||||||||||||||||||
MT  ASLVKMGRLEGWEVFAKPKV
```

d) NDUAD vs NDUAD^{QTY}

```
1                                                              60
WT  AASKVKQDMPPPGGYGPIDYKRNLPRRGLSGYSMLAIGIGTLIYGHWSIMKWNRERRRLQ
    ||||||||||||||||||||||||||||*|||||*|*|*||**|||||*|||||||||||
MT  AASKVKQDMPPPGGYGPIDYKRNLPRRGQSGYSMQATGTGTQTYGHWSTMKWNRERRRLQ

61                                                             120
WT  IEDFEARIALLPLLQAETDRRTLQMLRENLEEEAIIMKDVPDWKVGESVFHTTRWVPPLI
    ||||||||||||||||||||||||||||||||||||||||||||||||||||||||||||
MT  IEDFEARIALLPLLQAETDRRTLQMLRENLEEEAIIMKDVPDWKVGESVFHTTRWVPPLI

121                                                            180
WT  GELYGLRTTEEALHASHGFMWYT
    |||||||||||||||||||||||
MT  GELYGLRTTEEALHASHGFMWYT
```

e) NDUB1 vs NDUB1QTY

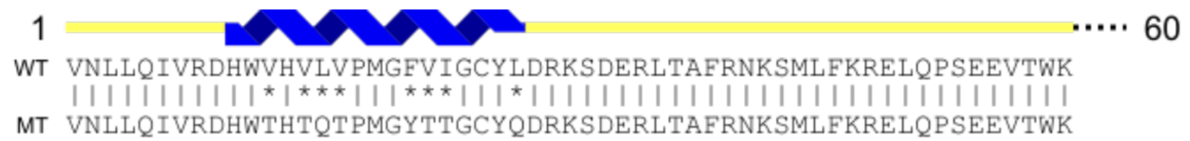

f) NDUB3 vs NDUB3QTY

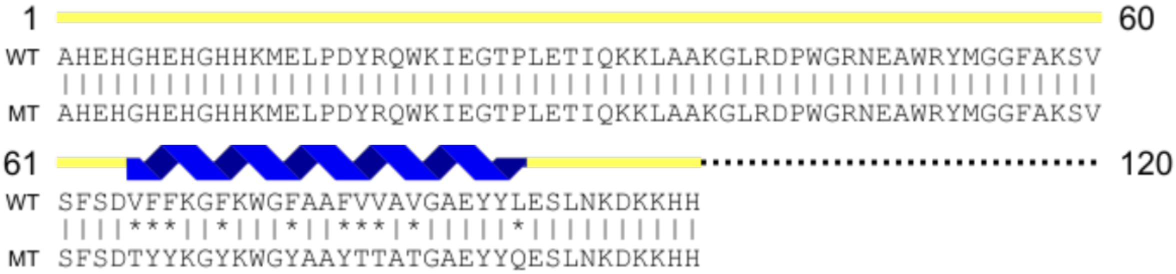

g) NDUB4 vs NDUB4QTY

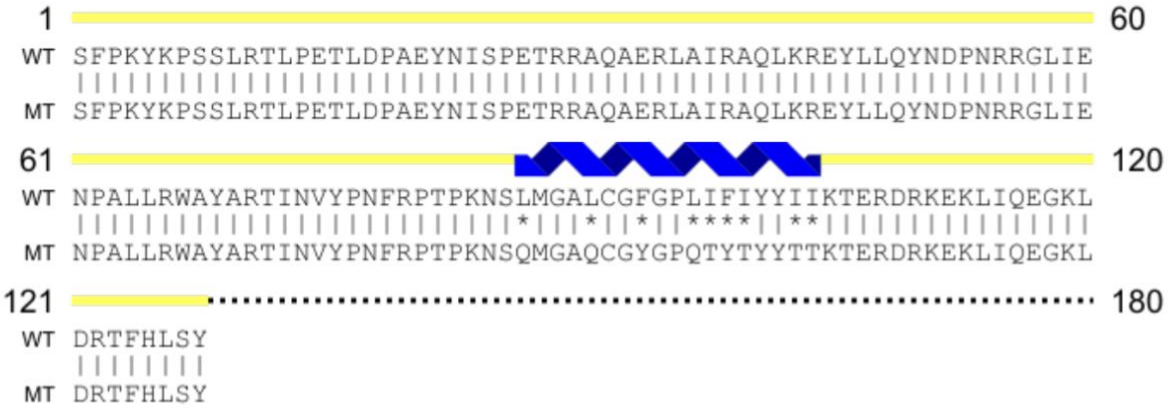

h) NDUB5 vs NDUB5QTY

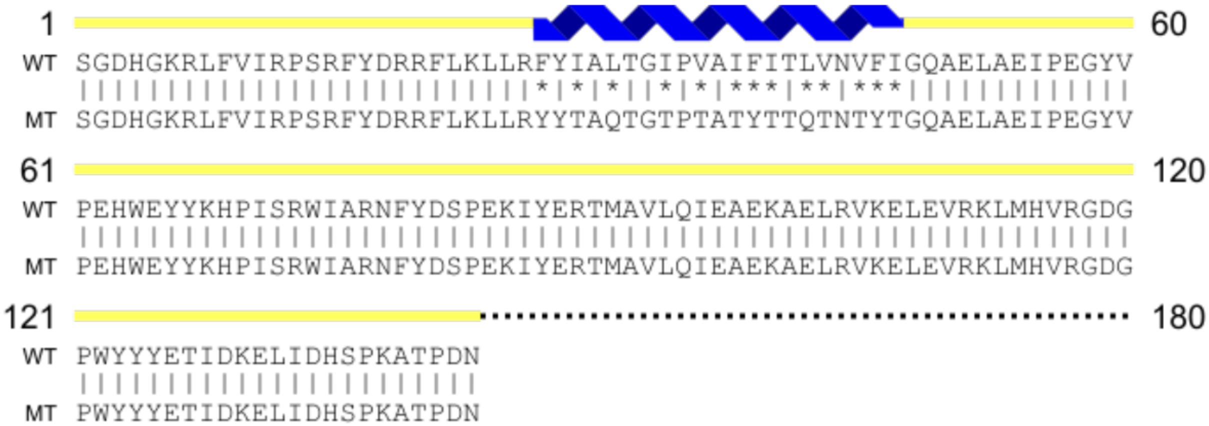

i) NDUB6 vs NDUB6^{QTY}

```
1                                                              60
WT TGYTPDEKLRLQQLRELRRRWLKDQELSPREPVLPPQKMGPMEKFWNKFLENKSPWRKMV
   ||||||||||||||||||||||||||||||||||||||||||||||||||||||||||||
MT TGYTPDEKLRLQQLRELRRRWLKDQELSPREPVLPPQKMGPMEKFWNKFLENKSPWRKMV

61                                                             120
WT HGVYKKSIFVFTHVLVPVWIIHYYMKYHVSEKPYGIVEKKSRIFPGDTILETGEVIPPMK
   |||||||****||***|*|**||||||||||||||||||||||||||||||||||||||
MT HGVYKKSTYTYTHTQTPTWTTHYYMKYHVSEKPYGIVEKKSRIFPGDTILETGEVIPPMK

121                                                            180
WT EFPDQHH
   |||||||
MT EFPDQHH
```

j) NDUB8 vs NDUB8^{QTY}

```
1                                                              60
WT ASHMTKDMFPGPYPRTPEERAAAAKKYNMRVEDYEPYPDDGMGYGDYPKLPDRSQHERDP
   ||||||||||||||||||||||||||||||||||||||||||||||||||||||||||||
MT ASHMTKDMFPGPYPRTPEERAAAAKKYNMRVEDYEPYPDDGMGYGDYPKLPDRSQHERDP

61                                                             120
WT WYSWDQPGLRLNWGEPMHWHLDMYNRNRVDTSPTPVSWHVMCMQLFGFLAFMIFMCWVGD
   ||||||||||||||||||||||||||||||||||||||||||||**|**|*|**|||*||
MT WYSWDQPGLRLNWGEPMHWHLDMYNRNRVDTSPTPVSWHVMCMQQYGYQAYMTYMCWTGD

121                                                            180
WT VYPVYQPVGPKQYPYNNLYLERGGDPSKEPERVVHYEI
   *||*||||||||||||||||||||||||||||||||||
MT TYPTYQPVGPKQYPYNNLYLERGGDPSKEPERVVHYEI
```

k) NDUBB vs NDUBB^{QTY}

```
1                                                              60
WT ESSFSRTVVAPSAVAGKRPPEPTTPWQEDPEPEDENLYEKNPDSHGYDKDPVLDVWNMRL
   |||||||||||||||||||||||||||||||||||||||||||||||||||||||||||*
MT ESSFSRTVVAPSAVAGKRPPEPTTPWQEDPEPEDENLYEKNPDSHGYDKDPVLDVWNMRQ

61                                                             120
WT VFFFGVSIILVLGSTFVAYLPDYRMKEWSRREAERLVKYREANGLPIMESNCFDPSKIQL
   ****|*|*****|||**||*||||||||||||||||||||||||||||||||||||||||
MT TYYYGTSTTQTQGSTYTAYQPDYRMKEWSRREAERLVKYREANGLPIMESNCFDPSKIQL

121                                                            180
WT PEDE
   ||||
MT PEDE
```

l) NDUC1 vs NDUC1[QTY]

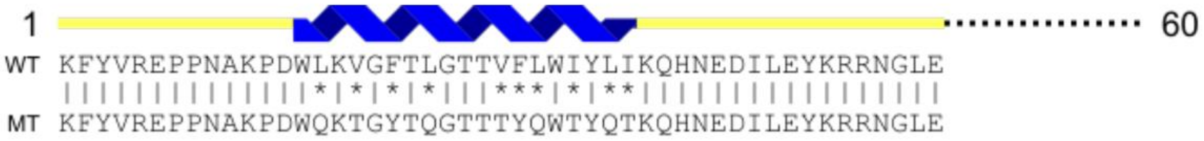

m) NDUC2 vs NDUC2[QTY]

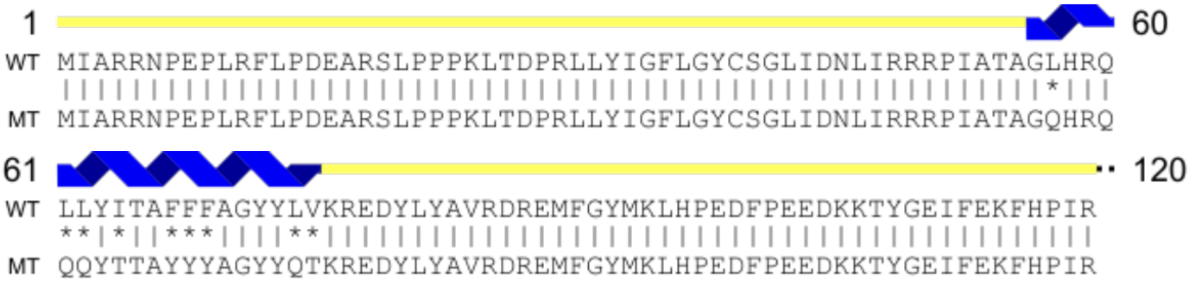

n) NU1M vs NU1M[QTY]

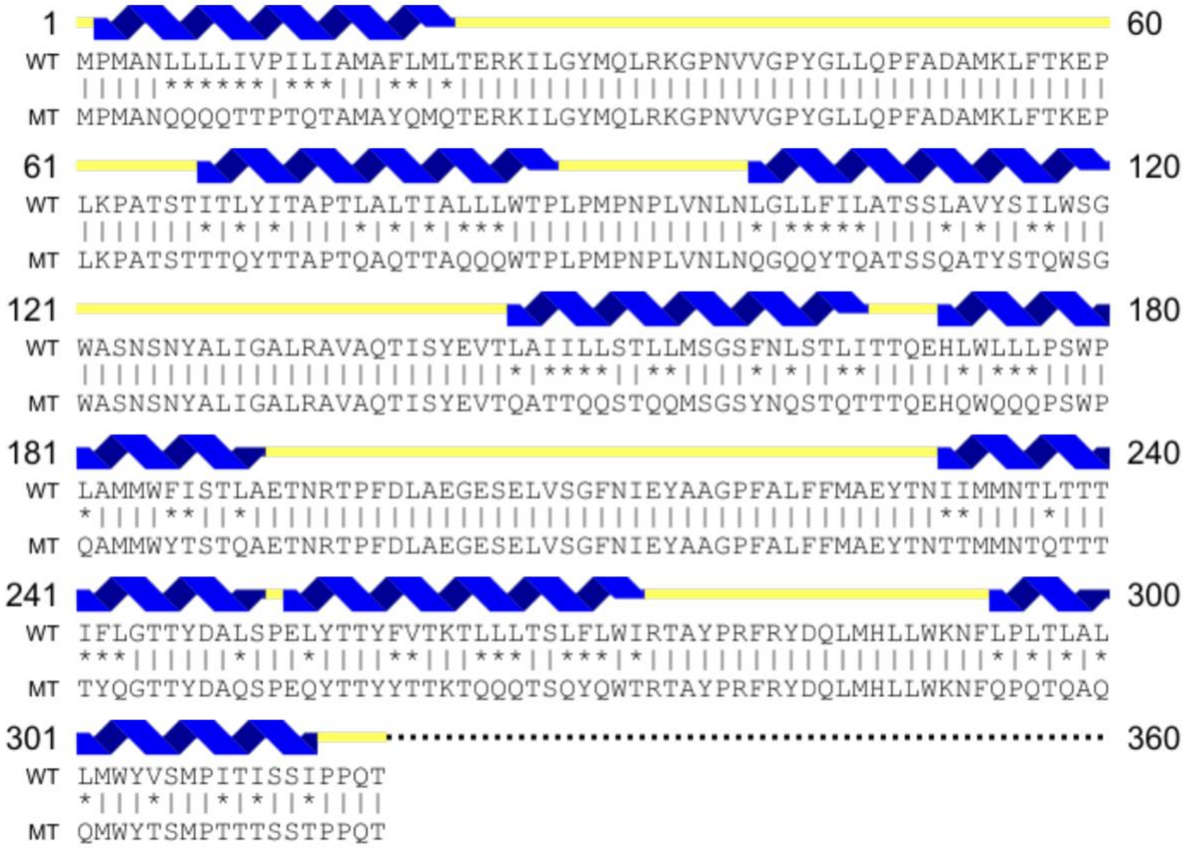

o) NU2M vs NU2M^{QTY}

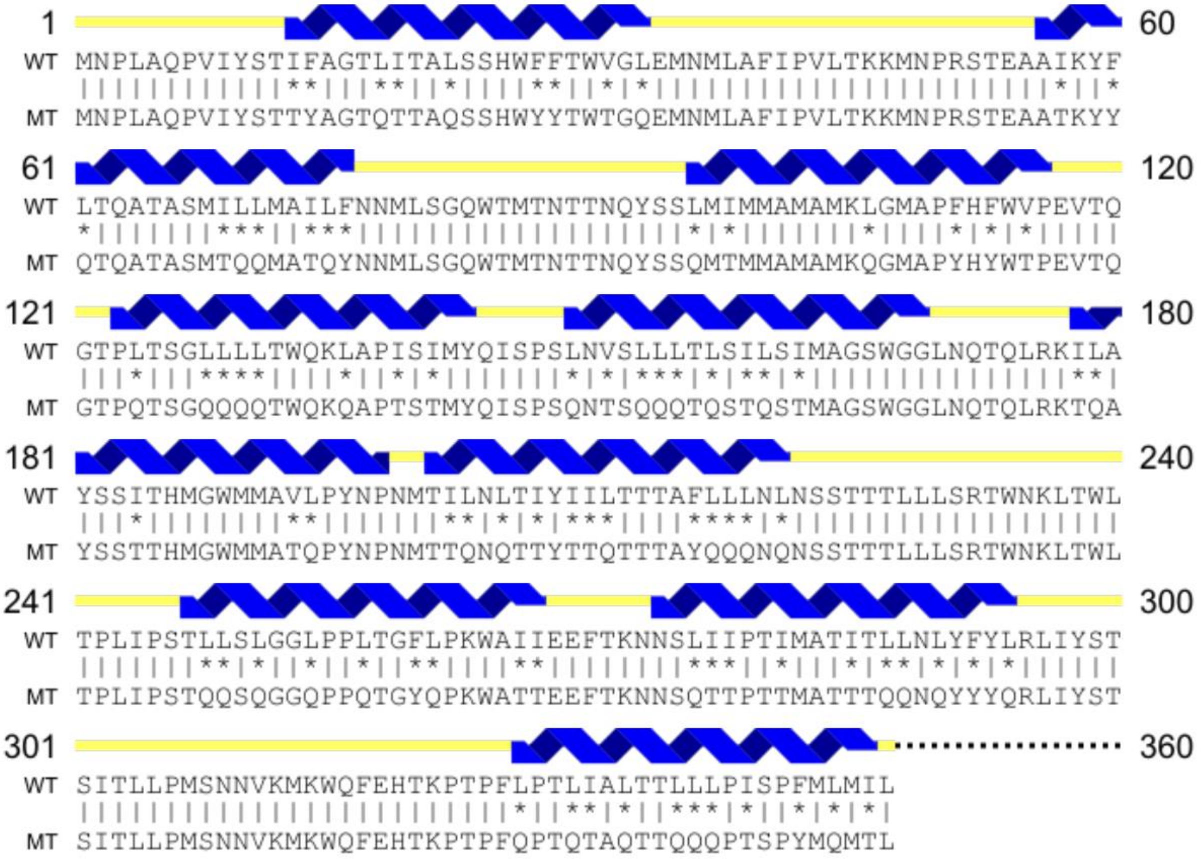

p) NU3M vs NU3M^{QTY}

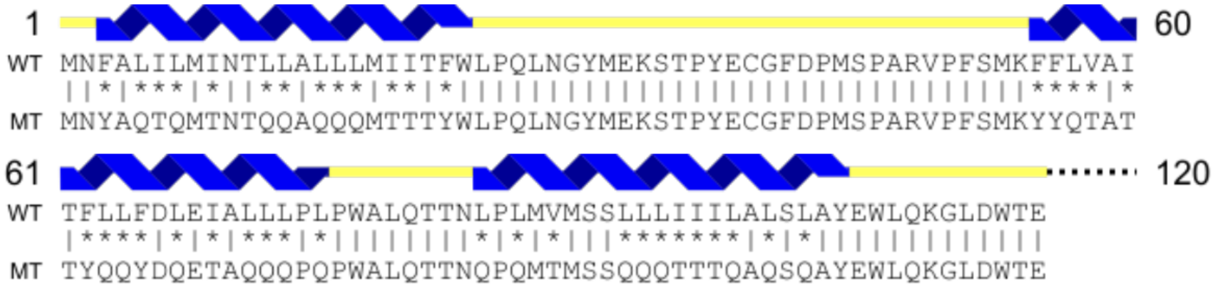

q) NU4M vs NU4M^{QTY}

```
1                                                                     60
WT MLKLIVPTIMLLPLTWLSKKHMIWINTTTHSLIISIIPLLFFNQINNNLFSCSPTFSSDP
   ||||||||||||||||||||||*|*||||||***|**|****||||||||||||||||||
MT MLKLIVPTIMLLPLTWLSKKHMTWTNTTTHSQTTSTTPQQYYNQINNNLFSCSPTFSSDP

61                                                                   120
WT LTTPLLMLTTWLLPLTIMASQRHLSSEPLSRKKLYLSMLISLQISLIMTFTATELIMFYI
   *|||**|*|||**|*|*||||||||||||||||||||||||*|*|**||*||||**|*|*
MT QTTPQQMQTTWQQPQTTMASQRHLSSEPLSRKKLYLSMLISQQTSQTMTYTATEQTMYYT

121                                                                  180
WT FFETTLIPTLAIITRWGNQPERLNAGTYFLFYTLVGSLPLLIALIYTHNTLGSLNILLLT
   **||||||||||||||||||||||||||***||**||*|***|**|||||||||||||||
MT YYETTLIPTLAIITRWGNQPERLNAGTYYQYYTQTGSQPQQTAQTYTHNTLGSLNILLLT

181                                                                  240
WT LTAQELSNSWANNLMWLAYTMAFMVKMPLYGLHLWLPKAHVEAPIAGSMVLAAVLLKLGG
   ||||||||||||||||*|||||*|*|||*||*|*|*||||||||*||||**||***|*||
MT LTAQELSNSWANNLMWQAYTMAYMTKMPQYGQHQWQPKAHVEAPTAGSMTQAATQQKQGG

241                                                                  300
WT YGMMRLTLILNPLTKHMAYPFLVLSLWGMIMTSSICLRQTDLKSLIAYSSISHMALVVTA
   ||||||||||||||||||||****|*|||*||||*|*|||||||**||||*||||***||
MT YGMMRLTLILNPLTKHMAYPYQTQSQWGMTMTSSTCQRQTDLKSQTAYSSTSHMAQTTTA

301                                                                  360
WT ILIQTPWSFTGAVILMIAHGLTSSLLFCLANSNYERTHSRIMILSQGLQTLLPLMAFWWL
   ***|||||*|||***|*|||*|||***|*|||||||||||||||||||||**|*||*||*
MT TQTQTPWSYTGATTQMTAHGQTSSQQYCQANSNYERTHSRIMILSQGLQTQQPQMAYWWQ

361                                                                  420
WT LASLANLALPPTINLLGELSVLVTTFSWSNITLLLTGLNMLVTALYSLYMFTTTQWGSLT
   *||*||*|*|||||||||||||||||||||*|***||*||**||*||*||*|||||||||
MT QASQANQAQPPTINLLGELSVLVTTFSWSNTTQQQTGQNMQTTAQYSQYMYTTTQWGSLT

421                                                                  480
WT HHINNMKPSFTRENTLMFMHLSPILLLSLNPDIITGFSS
   |||||||||||||||||||||||||||||||||||||||
MT HHINNMKPSFTRENTLMFMHLSPILLLSLNPDIITGFSS
```

r) NU5M vs NU5M^{QTY}

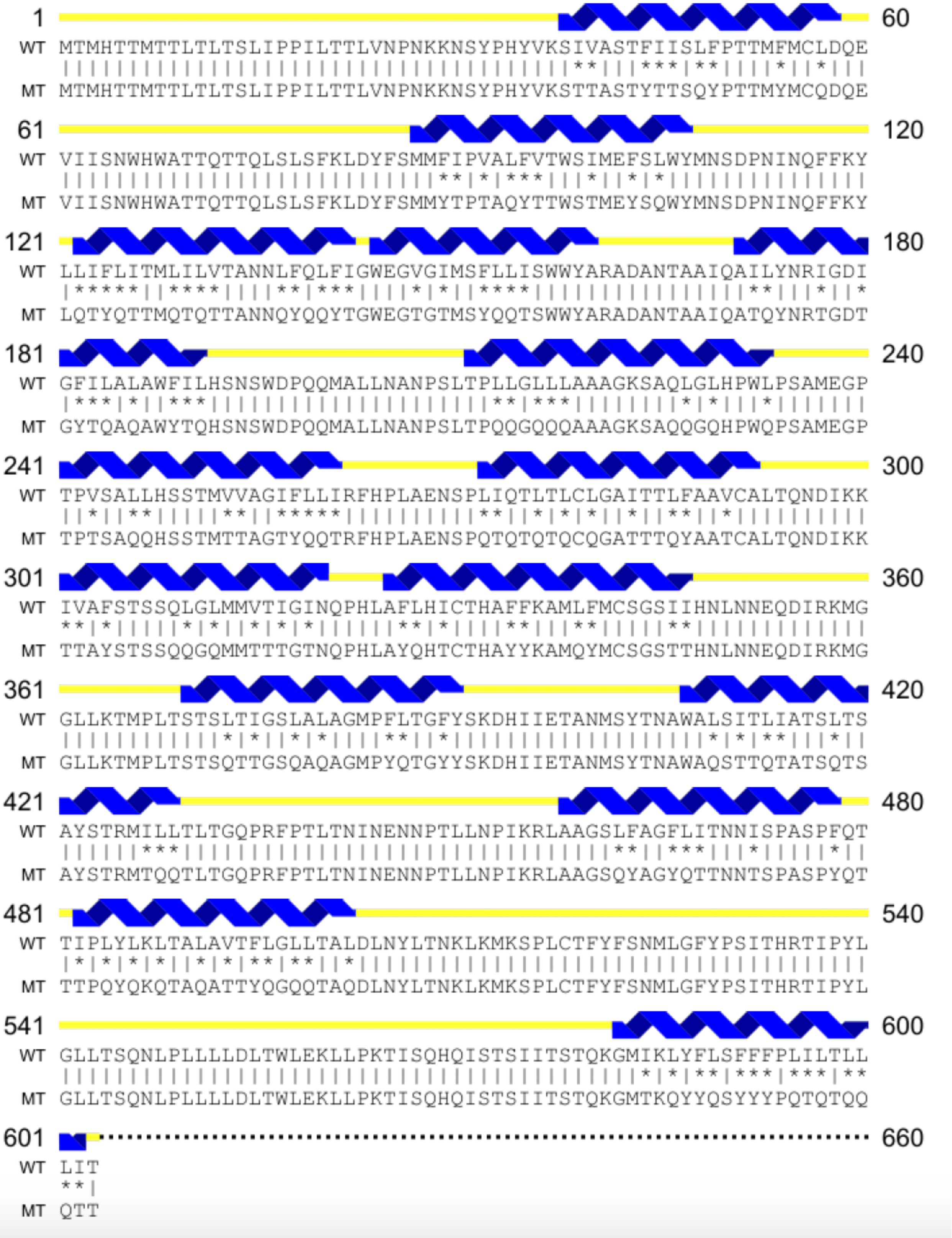

s) NU6M vs NU6M^{QTY}

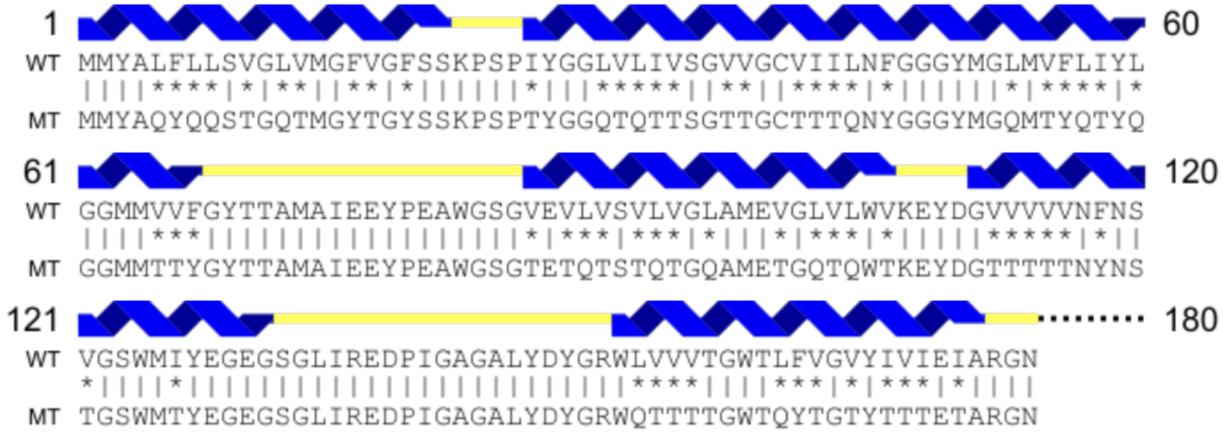

t) NU4LM vs NU4LM^{QTY}

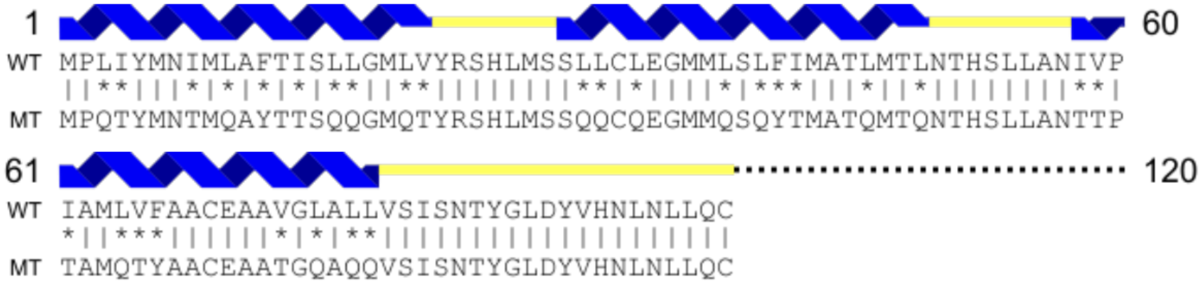

Figure S2. AlphaFlold 3 Prediction accuracy: pLDDT and PAE scores.

a) NDUA1^{QTY}

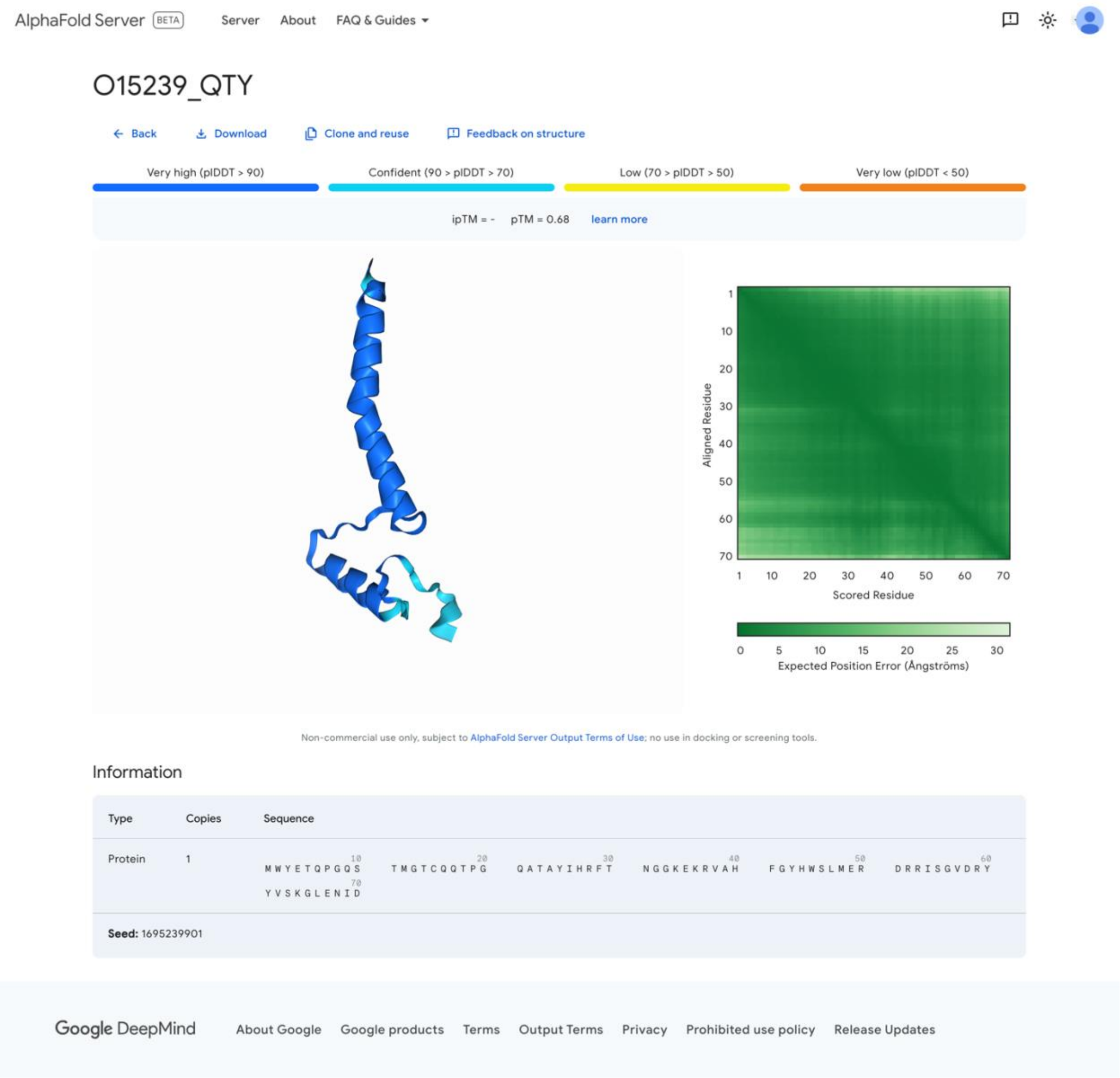

b) NDUA3^{QTY}

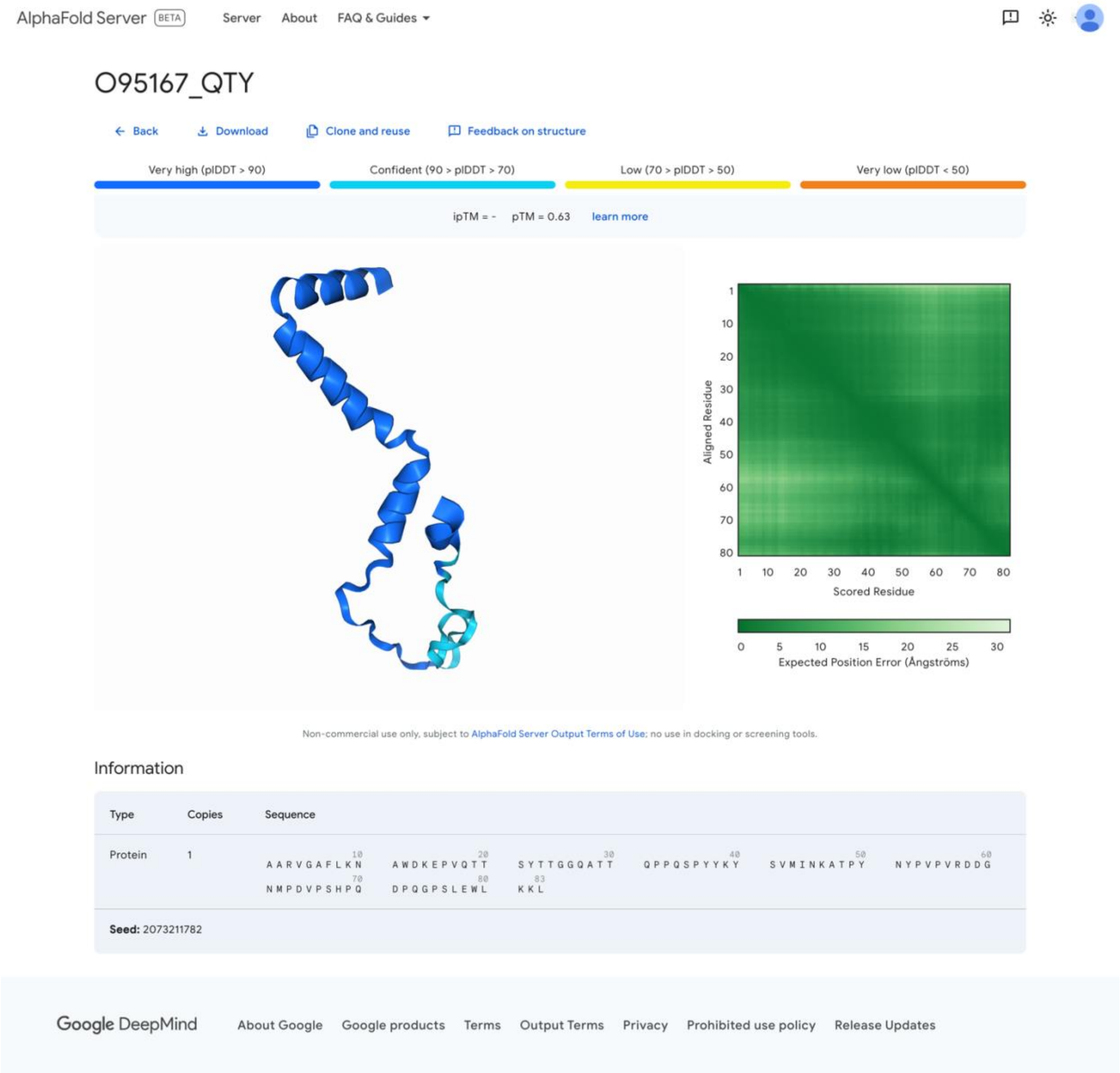

AlphaFold Server BETA
Server
About
FAQ & Guides
O95167_QTY
Back
Download
Clone and reuse
Feedback on structure
Very high (pIDDT > 90)
Confident (90 > pIDDT > 70)
Low (70 > pIDDT > 50)
Very low (pIDDT < 50)
ipTM = - pTM = 0.63 learn more
Aligned Residue
1 10 20 30 40 50 60 70 80
Scored Residue
0 5 10 15 20 25 30
Expected Position Error (Ångströms)
Non-commercial use only, subject to AlphaFold Server Output Terms of Use; no use in docking or screening tools.
Information
Type Copies Sequence
Protein 1
AARVGAFLKN AWDKEPVQTT SYTTGGQATT QPPQSPYYKY SVMINKATPY NYPVPVRDDG
NMPDVPSHPQ DPQGPSLEWL KKL
Seed: 2073211782
Google DeepMind
About Google
Google products
Terms
Output Terms
Privacy
Prohibited use policy
Release Updates

c) NDUAB^{QTY}

AlphaFold Server BETA   Server   About   FAQ & Guides

Q86Y39_QTY

Back   Download   Clone and reuse   Feedback on structure

Very high (pLDDT > 90)   Confident (90 > pLDDT > 70)   Low (70 > pLDDT > 50)   Very low (pLDDT < 50)

ipTM = -   pTM = 0.47   learn more

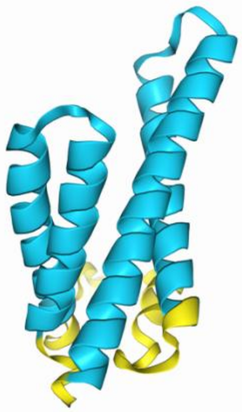

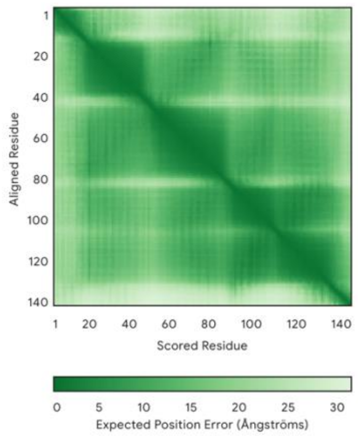

Non-commercial use only, subject to AlphaFold Server Output Terms of Use; no use in docking or screening tools.

Information

Type	Copies	Sequence
Protein	1	APKVFRQYWD IPDGTDCHRK AYSTTSTAST AGQTAAAYRT TQNPPGTFLE GVAKVGQYTY TAAATGATYG QTTCTSAHTR EKPDDPLNYF LGGCAGGLTL GARTHNYGIG AAACVYFGIA ASLVKMGRLE GWEVFAKPKV

Seed: 1336344939

Google DeepMind   About Google   Google products   Terms   Output Terms   Privacy   Prohibited use policy   Release Updates

d) NDUAD^{QTY}

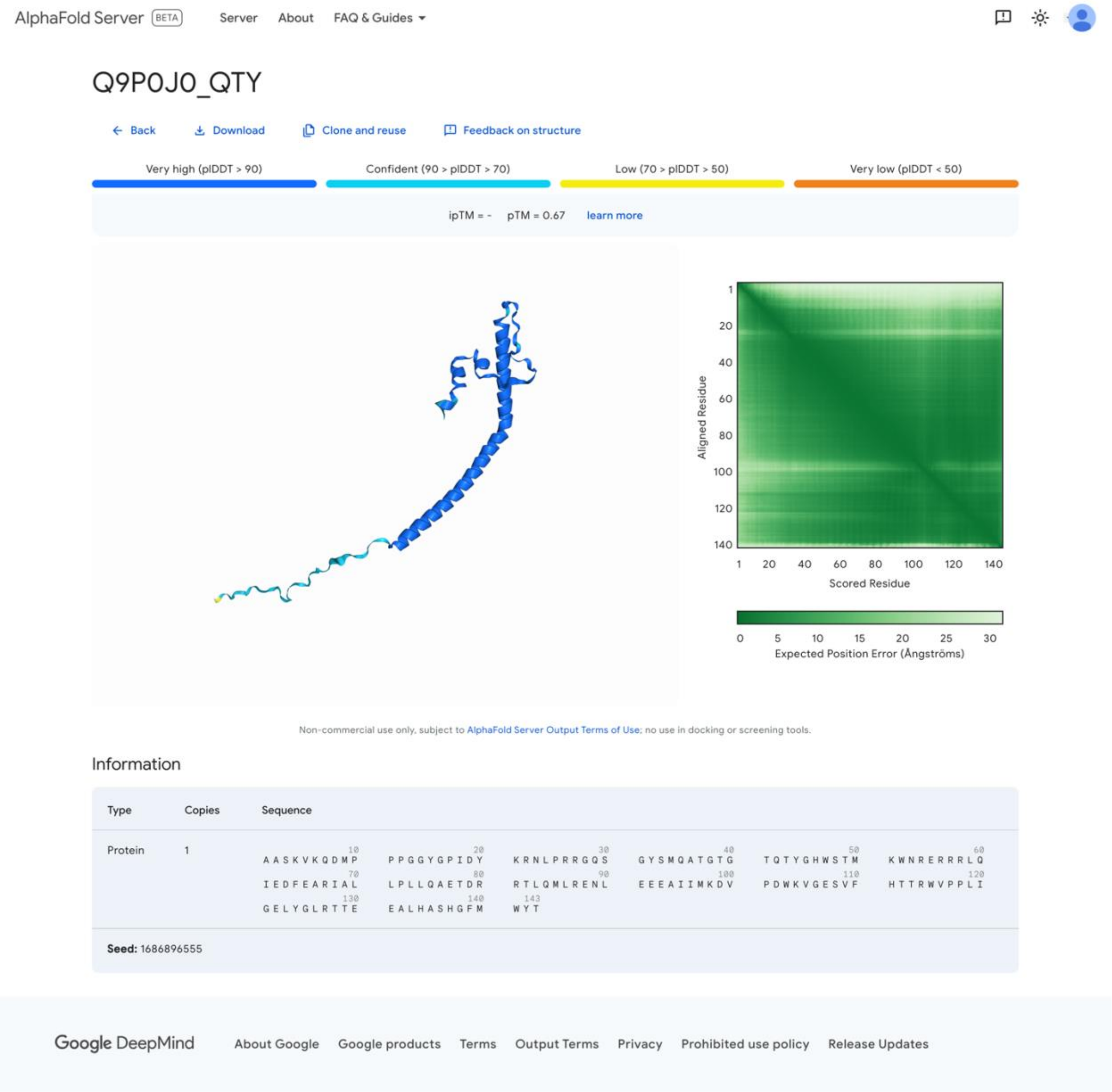
AlphaFold Server BETA
Server
About
FAQ & Guides
Q9P0J0_QTY
Back
Download
Clone and reuse
Feedback on structure
Very high (pIDDT > 90)
Confident (90 > pIDDT > 70)
Low (70 > pIDDT > 50)
Very low (pIDDT < 50)
ipTM = - pTM = 0.67
learn more
Aligned Residue
Scored Residue
Expected Position Error (Ångströms)
Non-commercial use only, subject to AlphaFold Server Output Terms of Use; no use in docking or screening tools.
Information
Type
Copies
Sequence
Protein
1
AASKVKQDMP PPGGYGPIDY KRNLPRRGQS GYSMQATGTG TQTYGHWSTM KWNRERRRLQ
IEDFEARIAL LPLLQAETDR RTLQMLRENL EEEAIIMKDV PDWKVGESVF HTTRWVPPLI
GELYGLRTTE EALHASHGFM WYT
Seed: 1686896555
Google DeepMind
About Google
Google products
Terms
Output Terms
Privacy
Prohibited use policy
Release Updates

e) NDUB1^{QTY}

AlphaFold Server BETA   Server   About   FAQ & Guides

O15239_TRY2

Back   Download   Clone and reuse   Feedback on structure

Very high (pLDDT > 90)	Confident (90 > pLDDT > 70)	Low (70 > pLDDT > 50)	Very low (pLDDT < 50)

ipTM = -   pTM = 0.43   learn more

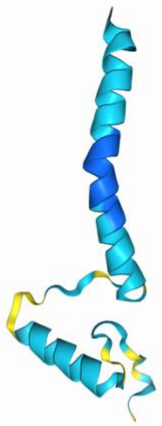

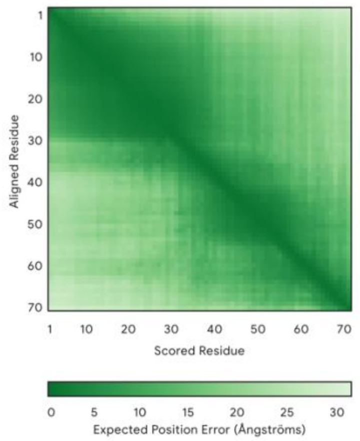

Non-commercial use only, subject to AlphaFold Server Output Terms of Use; no use in docking or screening tools.

Information

Type	Copies	Sequence
Protein	1	MWFEILPGLS VMGVCLLIPG LATAYIHRFT NGGKEKRVAH FGYHWSLMER DRRISGVDRY YVSKGLENID

Seed: 666879867

Google DeepMind   About Google   Google products   Terms   Output Terms   Privacy   Prohibited use policy   Release Updates

f) NDUB3^{QTY}

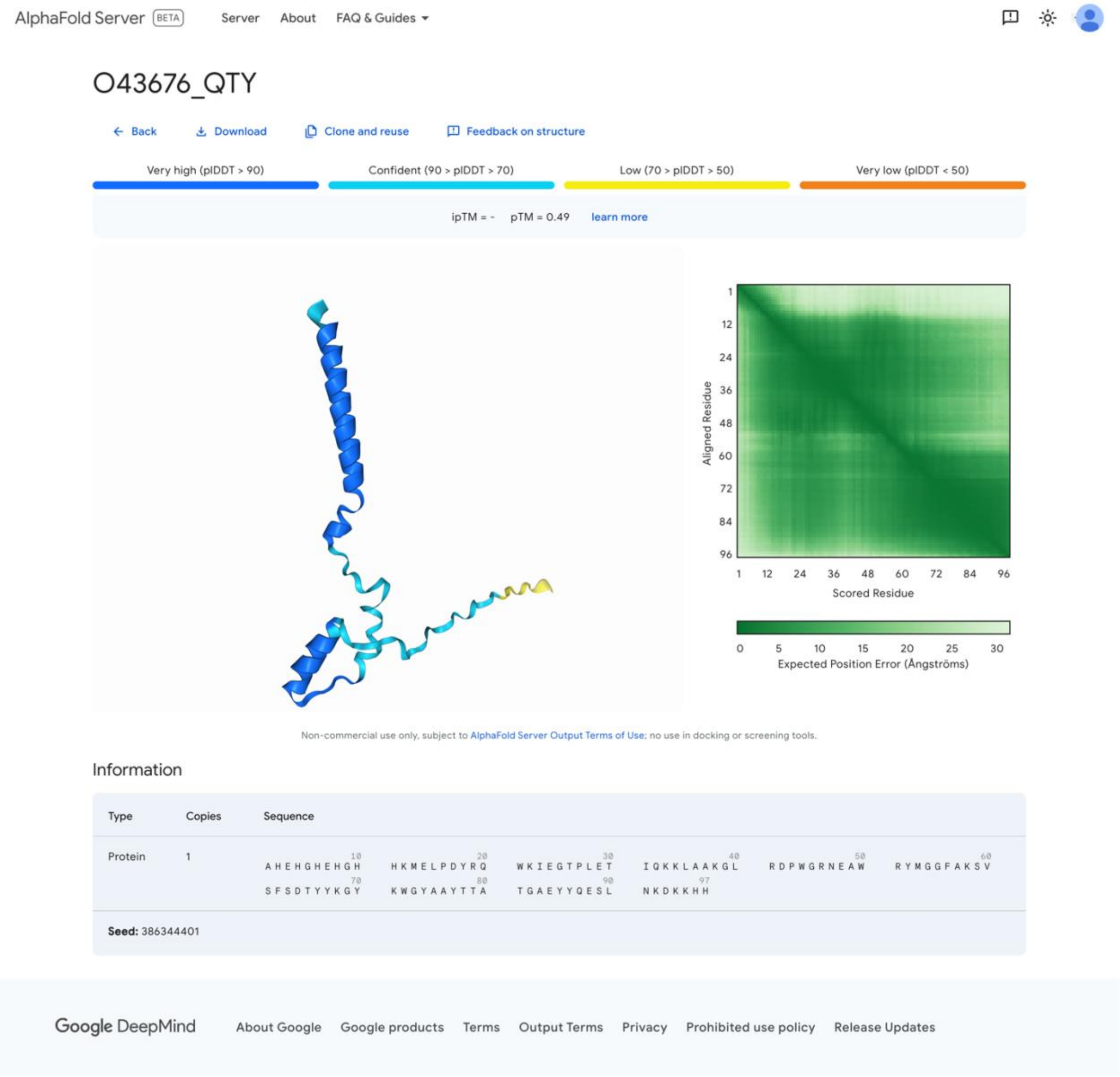

AlphaFold Server BETA
Server
About
FAQ & Guides
O43676_QTY
Back
Download
Clone and reuse
Feedback on structure
Very high (pIDDT > 90)
Confident (90 > pIDDT > 70)
Low (70 > pIDDT > 50)
Very low (pIDDT < 50)
ipTM = - pTM = 0.49 learn more
Aligned Residue
Scored Residue
1 12 24 36 48 60 72 84 96
0 5 10 15 20 25 30
Expected Position Error (Ångströms)
Non-commercial use only, subject to AlphaFold Server Output Terms of Use; no use in docking or screening tools.
Information
Type
Copies
Sequence
Protein
1
AHEHGHEHGH HKMELPDYRQ WKIEGTPLET IQKKLAAKGL RDPWGRNEAW RYMGGFAKSV
SFSDTYYKGY KWGYAAYTTA TGAEYYQESL NKDKKHH
Seed: 386344401
Google DeepMind
About Google
Google products
Terms
Output Terms
Privacy
Prohibited use policy
Release Updates

g) NDUB4^{QTY}

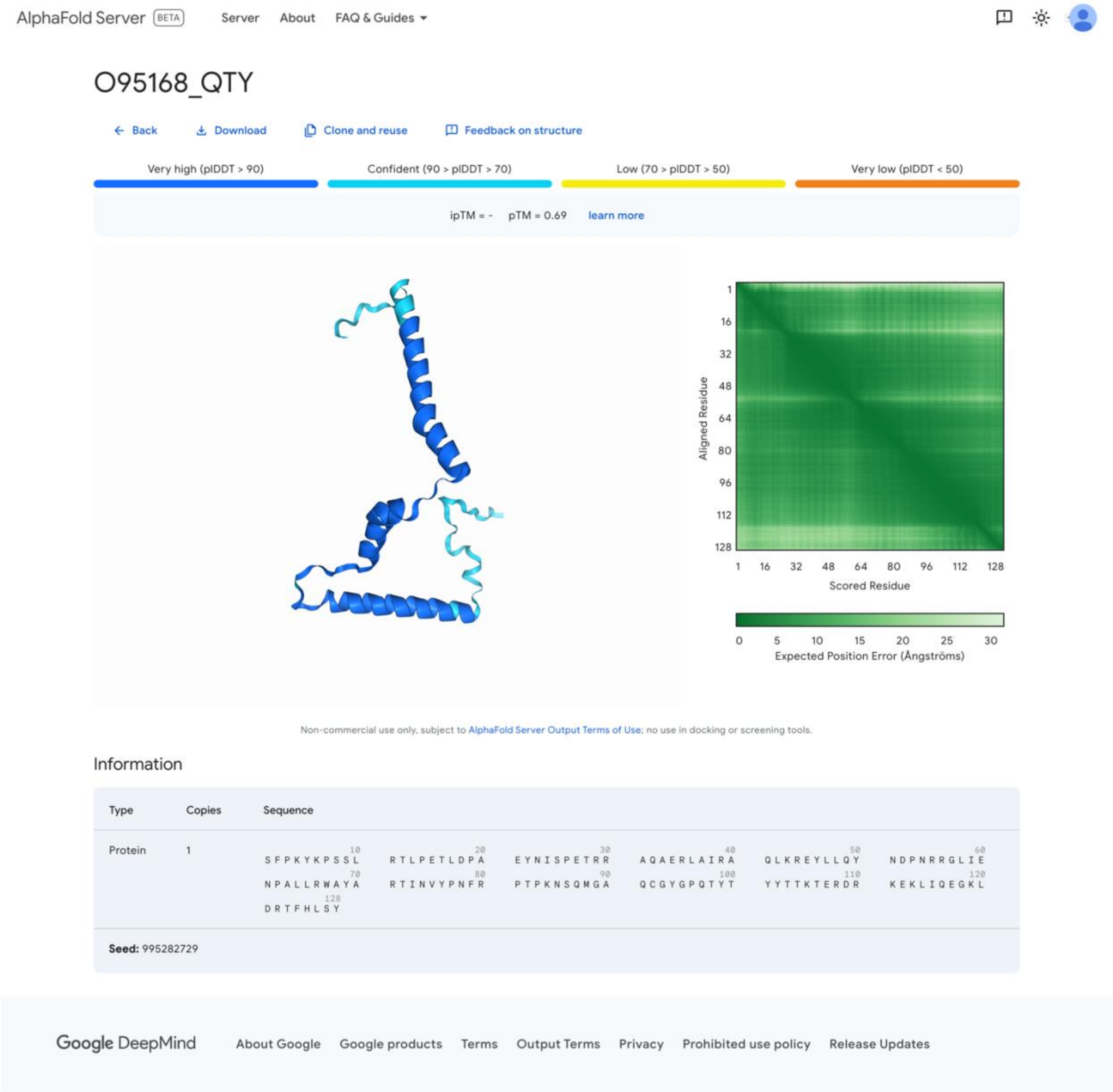

AlphaFold Server BETA
Server
About
FAQ & Guides
O95168_QTY
Back
Download
Clone and reuse
Feedback on structure
Very high (pIDDT > 90)
Confident (90 > pIDDT > 70)
Low (70 > pIDDT > 50)
Very low (pIDDT < 50)
ipTM = -   pTM = 0.69
learn more
Aligned Residue
1 16 32 48 64 80 96 112 128
Scored Residue
0 5 10 15 20 25 30
Expected Position Error (Ångströms)
Non-commercial use only, subject to AlphaFold Server Output Terms of Use; no use in docking or screening tools.
Information
Type
Copies
Sequence
Protein
1
SFPKYKPSSL RTLPETLDPA EYNISPETRR AQAERLAIRA QLKREYLLQY NDPNRRGLIE
NPALLRWAYA RTINVYPNFR PTPKNSQMGA QCGYGPQTYT YYTTKTERDR KEKLIQEGKL
DRTFHLSY
Seed: 995282729
Google DeepMind
About Google
Google products
Terms
Output Terms
Privacy
Prohibited use policy
Release Updates

h) NDUB5^{QTY}

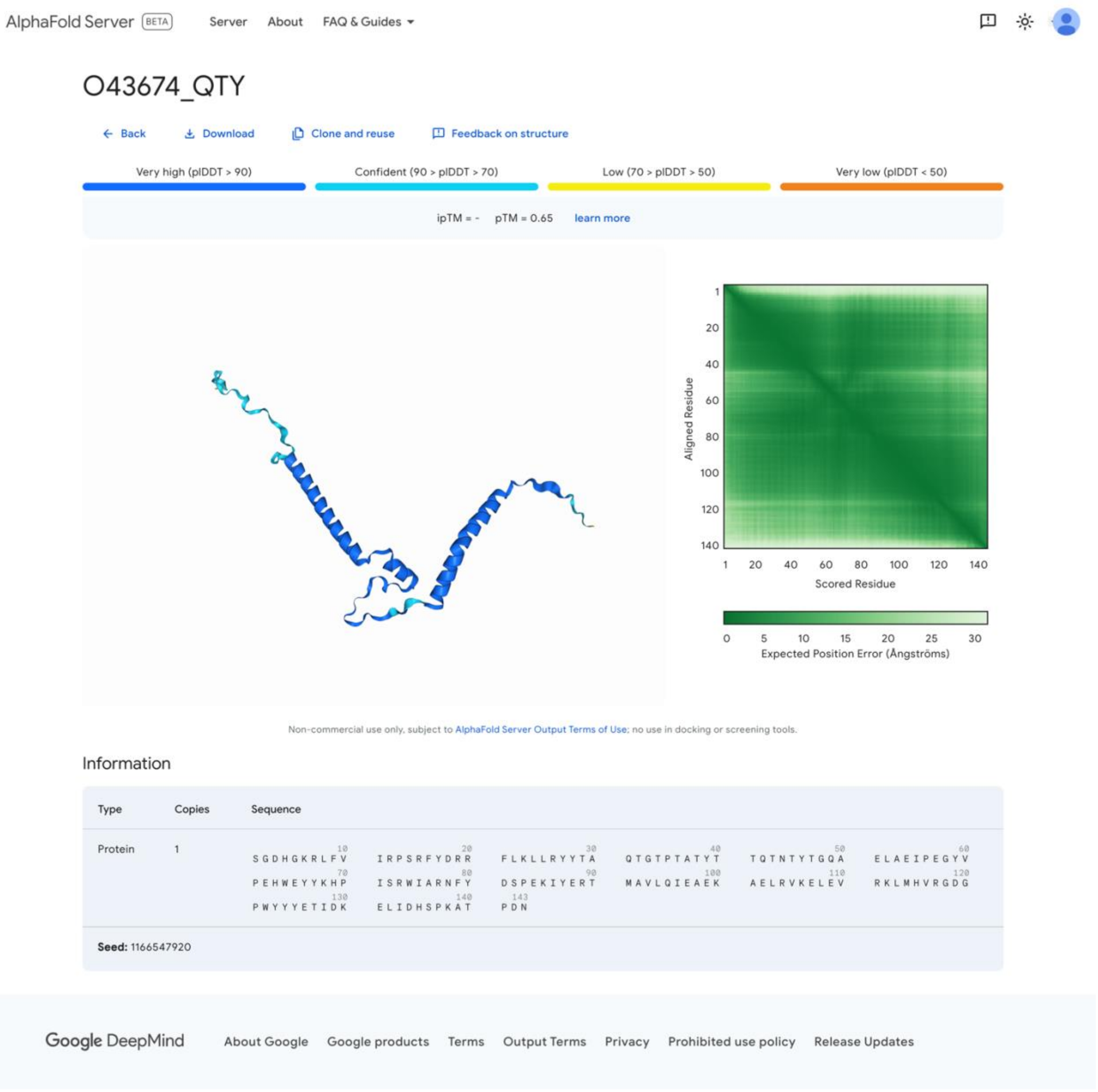
AlphaFold Server BETA
Server
About
FAQ & Guides
O43674_QTY
Back
Download
Clone and reuse
Feedback on structure
Very high (pIDDT > 90)
Confident (90 > pIDDT > 70)
Low (70 > pIDDT > 50)
Very low (pIDDT < 50)
ipTM = - pTM = 0.65
learn more
Aligned Residue
Scored Residue
Expected Position Error (Ångströms)
Non-commercial use only, subject to AlphaFold Server Output Terms of Use; no use in docking or screening tools.
Information
Type
Copies
Sequence
Protein
1
SGDHGKRLFV IRPSRFYDRR FLKLLRYYTA QTGTPTATYT TQTNTYTGQA ELAEIPEGYV
PEHWEYYKHP ISRWIARNFY DSPEKIYERT MAVLQIEAEK AELRVKELEV RKLMHVRGDG
PWYYYETIDK ELIDHSPKAT PDN
Seed: 1166547920
Google DeepMind
About Google
Google products
Terms
Output Terms
Privacy
Prohibited use policy
Release Updates

i) NDUB6^{QTY}

AlphaFold Server BETA Server About FAQ & Guides

O95139_QTY

Back Download Clone and reuse Feedback on structure

Very high (pLDDT > 90) | Confident (90 > pLDDT > 70) | Low (70 > pLDDT > 50) | Very low (pLDDT < 50)

ipTM = - pTM = 0.45 learn more

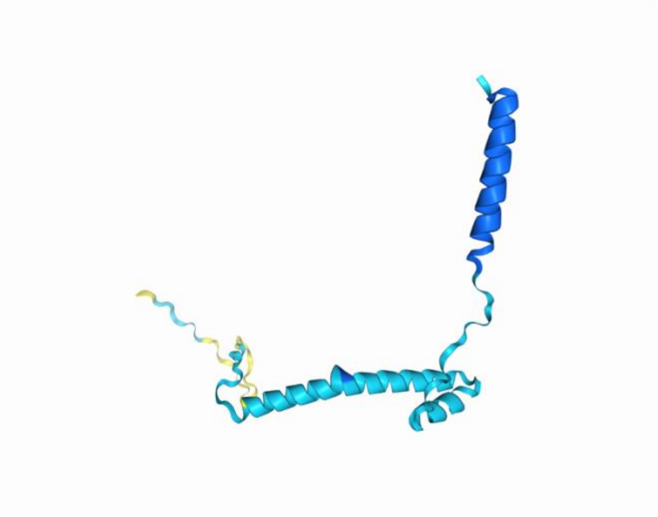

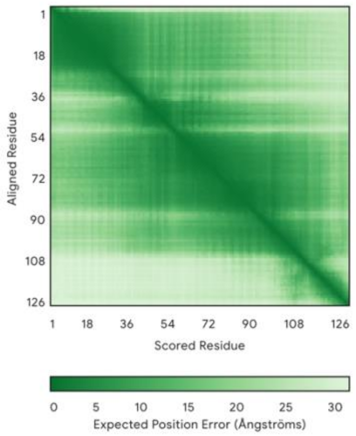

Non-commercial use only, subject to AlphaFold Server Output Terms of Use; no use in docking or screening tools.

Information

Type	Copies	Sequence
Protein	1	TGYTPDEKLR LQQLRELRRR WLKDQELSPR EPVLPPQKMG PMEKFWNKFL ENKSPWRKMV HGVYKKSTYT YTHTQTPTWT THYYMKYHVS EKPYGIVEKK SRIFPGDTIL ETGEVIPPMK EFPDQHH

Seed: 1766921277

Google DeepMind About Google Google products Terms Output Terms Privacy Prohibited use policy Release Updates

j) NDUB8^{QTY}

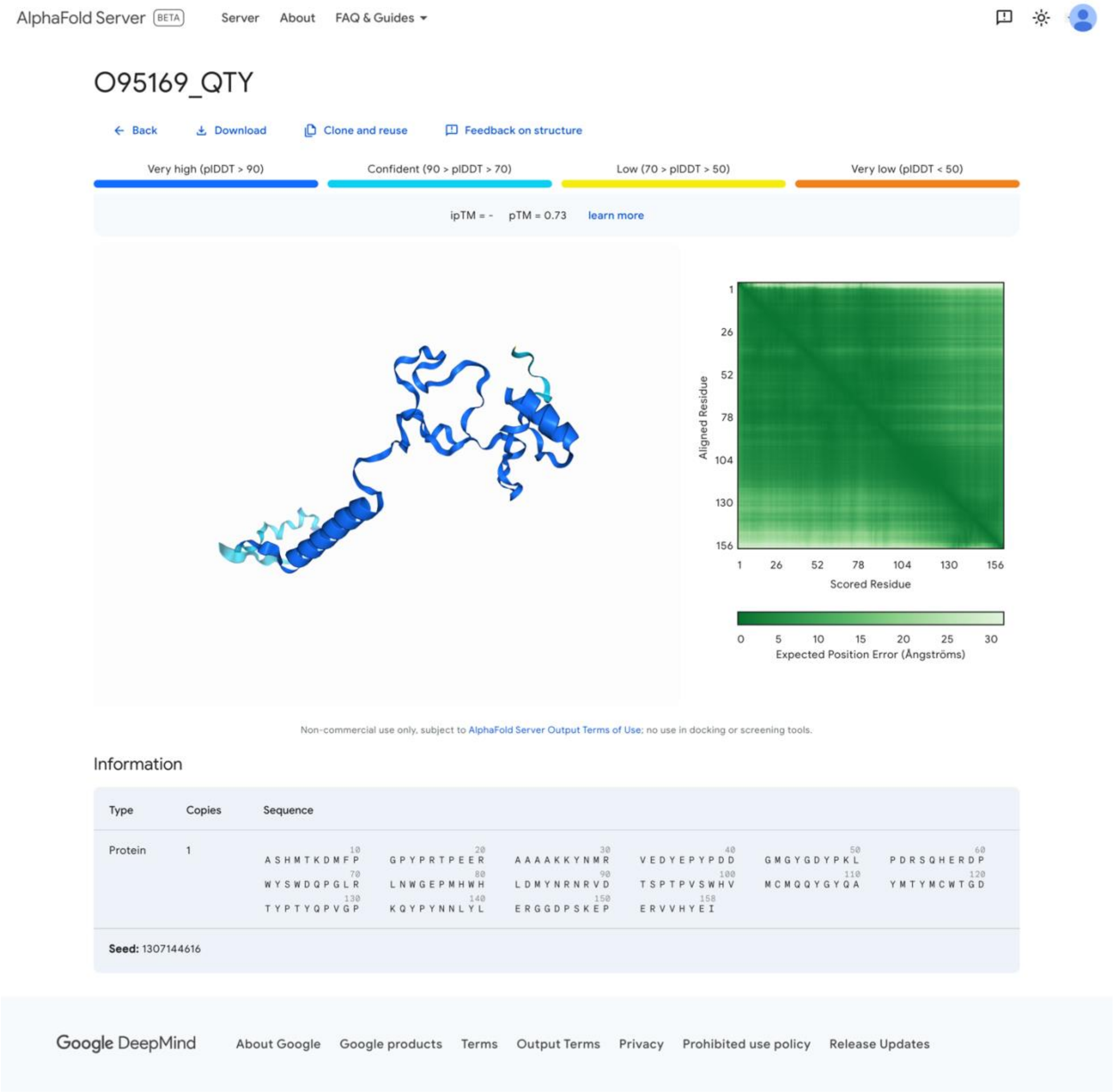
AlphaFold Server BETA
Server
About
FAQ & Guides
O95169_QTY
Back
Download
Clone and reuse
Feedback on structure
Very high (pIDDT > 90)
Confident (90 > pIDDT > 70)
Low (70 > pIDDT > 50)
Very low (pIDDT < 50)
ipTM = - pTM = 0.73 learn more
Aligned Residue
1 26 52 78 104 130 156
Scored Residue
1 26 52 78 104 130 156
0 5 10 15 20 25 30
Expected Position Error (Ångströms)
Non-commercial use only, subject to AlphaFold Server Output Terms of Use; no use in docking or screening tools.
Information
Type
Copies
Sequence
Protein
1
ASHMTKDMFP GPYPRTPEER AAAAKKYNMR VEDYEPYPDD GMGYGDYPKL PDRSQHERDP
WYSWDQPGLR LNWGEPMHWH LDMYNRNRVD TSPTPVSWHV MCMQQYGYQA YMTYMCWTGD
TYPTYQPVGP KQYPYNNLYL ERGGDPSKEP ERVVHYEI
10 20 30 40 50 60 70 80 90 100 110 120 130 140 150 158
Seed: 1307144616
Google DeepMind
About Google
Google products
Terms
Output Terms
Privacy
Prohibited use policy
Release Updates

k) NDUBB^{QTY}

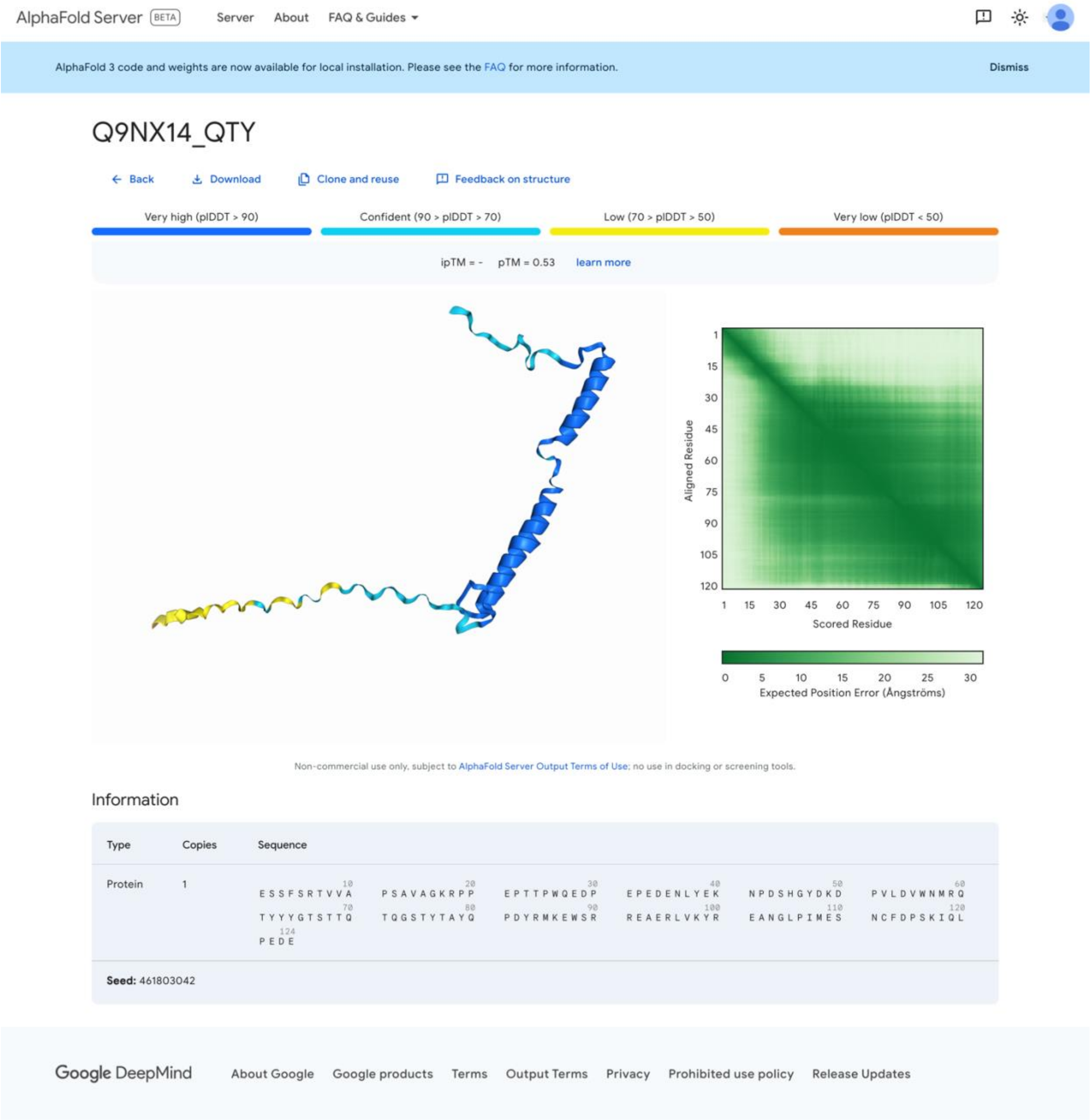

AlphaFold Server BETA
Server
About
FAQ & Guides
AlphaFold 3 code and weights are now available for local installation. Please see the FAQ for more information.
Dismiss
Q9NX14_QTY
Back
Download
Clone and reuse
Feedback on structure
Very high (pIDDT > 90)
Confident (90 > pIDDT > 70)
Low (70 > pIDDT > 50)
Very low (pIDDT < 50)
ipTM = - pTM = 0.53
learn more
Aligned Residue
1 15 30 45 60 75 90 105 120
Scored Residue
1 15 30 45 60 75 90 105 120
0 5 10 15 20 25 30
Expected Position Error (Ångströms)
Non-commercial use only, subject to AlphaFold Server Output Terms of Use; no use in docking or screening tools.
Information
Type
Copies
Sequence
Protein
1
ESSFSRTVVA PSAVAGKRPP EPTTPWQEDP EPEDENLYEK NPDSHGYDKD PVLDVWNMRQ
TYYYGTSTTQ TQGSTYTAYQ PDYRMKEWSR REAERLVKYR EANGLPIMES NCFDPSKIQL
PEDE
10 20 30 40 50 60 70 80 90 100 110 120 124
Seed: 461803042
Google DeepMind
About Google
Google products
Terms
Output Terms
Privacy
Prohibited use policy
Release Updates

I) NDUC1^{QTY}

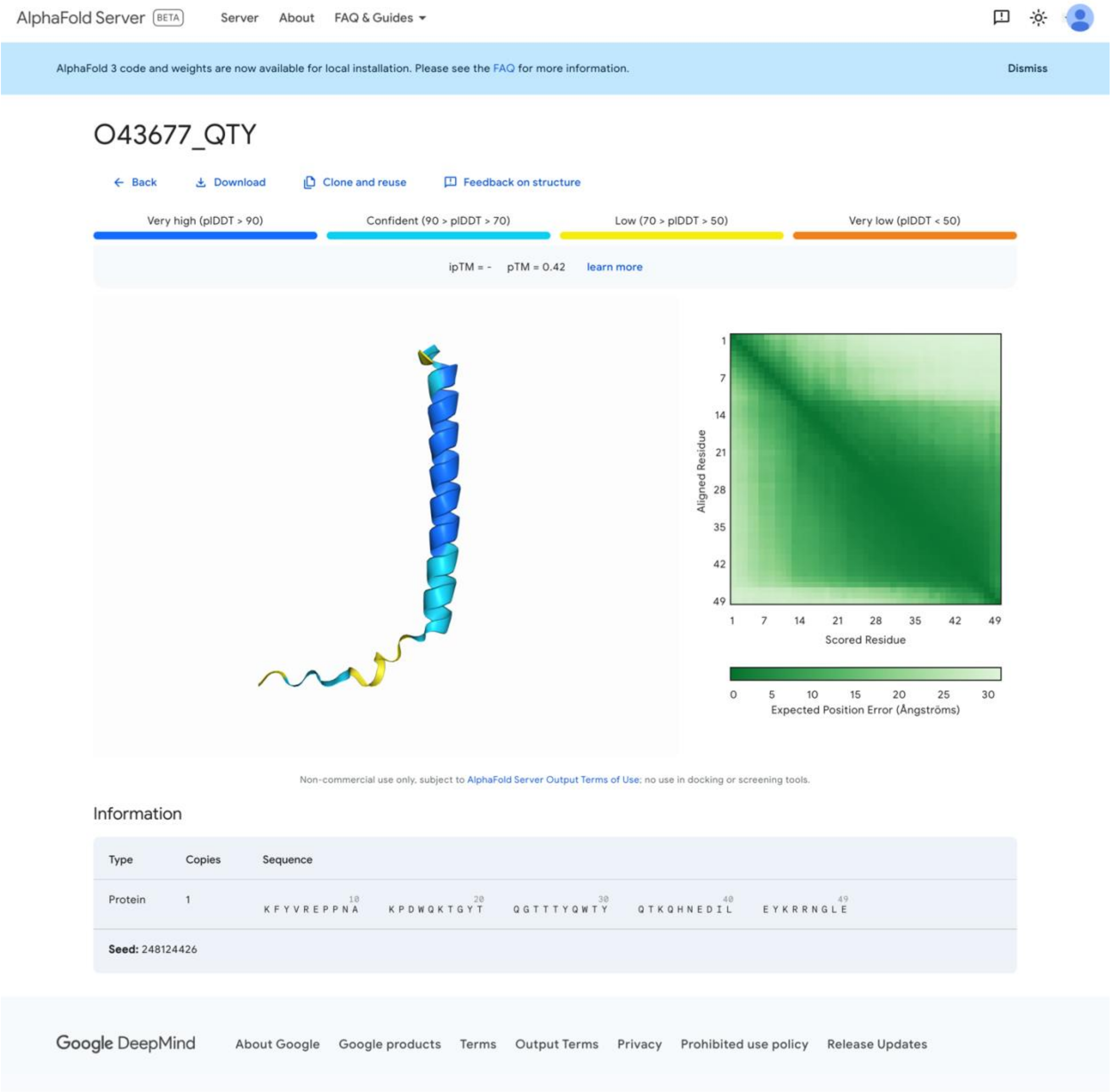

AlphaFold Server BETA
Server
About
FAQ & Guides
AlphaFold 3 code and weights are now available for local installation. Please see the FAQ for more information.
Dismiss
O43677_QTY
Back
Download
Clone and reuse
Feedback on structure
Very high (pIDDT > 90)
Confident (90 > pIDDT > 70)
Low (70 > pIDDT > 50)
Very low (pIDDT < 50)
ipTM = - pTM = 0.42 learn more
Aligned Residue
1 7 14 21 28 35 42 49
Scored Residue
0 5 10 15 20 25 30
Expected Position Error (Ångströms)
Non-commercial use only, subject to AlphaFold Server Output Terms of Use; no use in docking or screening tools.
Information
Type
Copies
Sequence
Protein
1
KFYVREPPNA KPDWQKTGYT QGTTTYQWTY QTKQHNEDIL EYKRRNGLE
10 20 30 40 49
Seed: 248124426
Google DeepMind
About Google
Google products
Terms
Output Terms
Privacy
Prohibited use policy
Release Updates

m) NDUC2^{QTY}

AlphaFold Server BETA | Server | About | FAQ & Guides

AlphaFold 3 code and weights are now available for local installation. Please see the FAQ for more information. Dismiss

O95298_QTY

← Back | Download | Clone and reuse | Feedback on structure

Very high (pIDDT > 90) | Confident (90 > pIDDT > 70) | Low (70 > pIDDT > 50) | Very low (pIDDT < 50)

ipTM = - pTM = 0.76 learn more

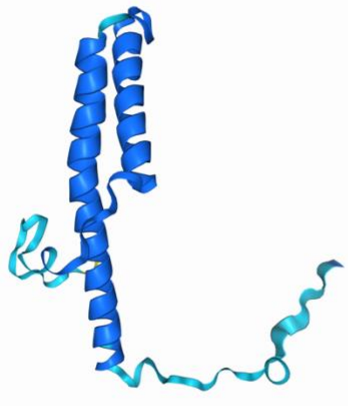

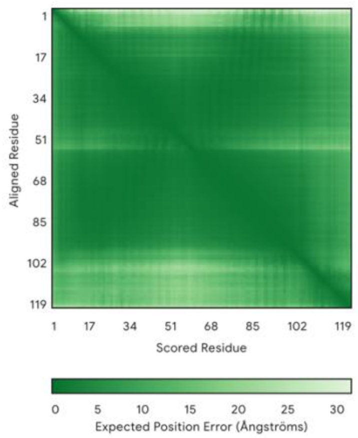

Non-commercial use only, subject to AlphaFold Server Output Terms of Use; no use in docking or screening tools.

Information

Type	Copies	Sequence
Protein	1	MIARRNPEPL[10] RFLPDEARSL[20] PPPKLTDPRL[30] LYIGFLGYCS[40] GLIDNLIRRR[50] PIATAGQHRQ[60] QQYTTAYYYA[70] GYYQTKREDY[80] LYAVRDREMF[90] GYMKLHPEDF[100] PEEDKKTYGE[110] IFEKFHPIR[119]

Seed: 643235458

Google DeepMind | About Google | Google products | Terms | Output Terms | Privacy | Prohibited use policy | Release Updates

n) NU1M^{QTY}

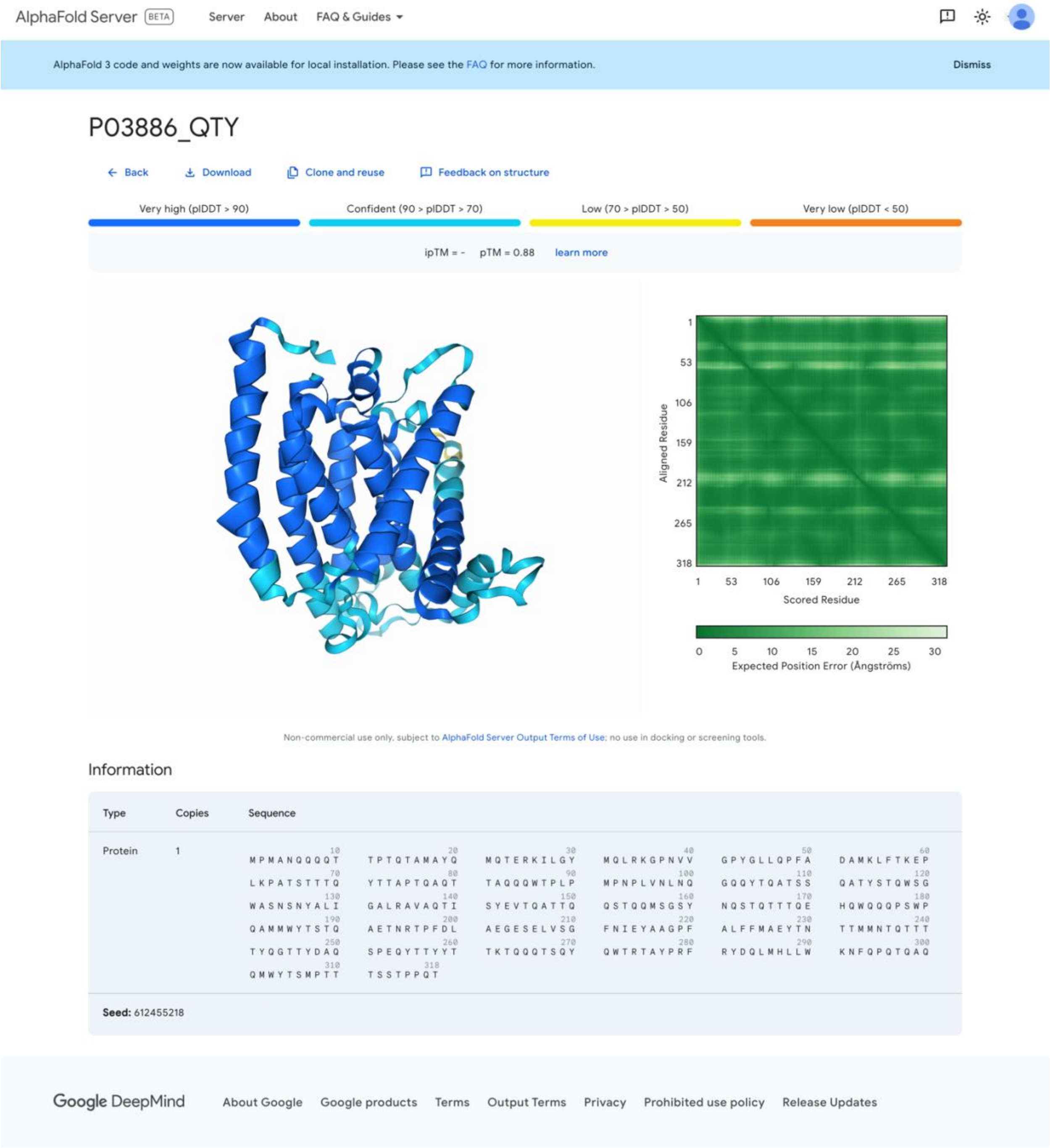

AlphaFold Server BETA
Server
About
FAQ & Guides
AlphaFold 3 code and weights are now available for local installation. Please see the FAQ for more information.
Dismiss
P03886_QTY
Back
Download
Clone and reuse
Feedback on structure
Very high (pIDDT > 90)
Confident (90 > pIDDT > 70)
Low (70 > pIDDT > 50)
Very low (pIDDT < 50)
ipTM = - pTM = 0.88
learn more
Aligned Residue
Scored Residue
1 53 106 159 212 265 318
0 5 10 15 20 25 30
Expected Position Error (Ångströms)
Non-commercial use only, subject to AlphaFold Server Output Terms of Use; no use in docking or screening tools.
Information
Type
Copies
Sequence
Protein
1
MPMANQQQQT TPTQTAMAYQ MQTERKILGY MQLRKGPNVV GPYGLLQPFA DAMKLFTKEP
LKPATSTTTQ YTTAPTQAQT TAQQQWTPLP MPNPLVNLNQ GQQYTQATSS QATYSTQWSG
WASNSNYALI GALRAVAQTI SYEVTQATTQ QSTQQMSGSY NQSTQTTTQE HQWQQQPSWP
QAMMWYTSTQ AETNRTPFDL AEGESELVSG FNIEYAAGPF ALFFMAEYTN TTMMNTQTTT
TYQGTTYDAQ SPEQYTTYYT TKTQQQTSQY QWTRTAYPRF RYDQLMHLLW KNFQPQTQAQ
QMWYTSMPTT TSSTPPQT
Seed: 612455218
Google DeepMind
About Google
Google products
Terms
Output Terms
Privacy
Prohibited use policy
Release Updates

o) NU2M^{QTY}

AlphaFold Server BETA Server About FAQ & Guides

AlphaFold 3 code and weights are now available for local installation. Please see the FAQ for more information. Dismiss

P03891_QTY

← Back | Download | Clone and reuse | Feedback on structure

Very high (pLDDT > 90) | Confident (90 > pLDDT > 70) | Low (70 > pLDDT > 50) | Very low (pLDDT < 50)

ipTM = - pTM = 0.93 learn more

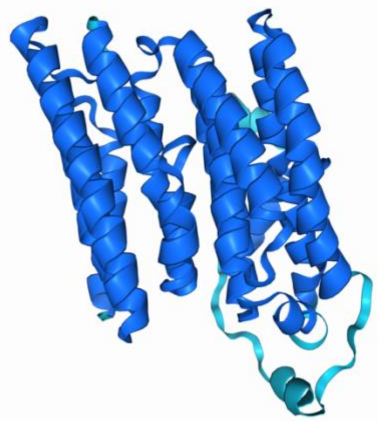

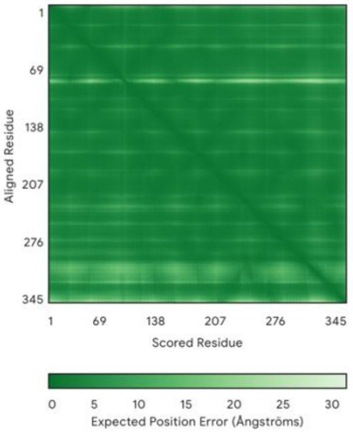

Non-commercial use only, subject to AlphaFold Server Output Terms of Use; no use in docking or screening tools.

Information

Type	Copies	Sequence
Protein	1	MNPLAQPVIY (10) STTYAGTQTT (20) AQSSHWYYTW (30) TGQEMNMLAF (40) IPVLTKKMNP (50) RSTEAATKYY (60) QTQATASMTQ (70) QMATQYNNML (80) SGQWTMTNTT (90) NQYSSQMTMM (100) AMAMKQGMAP (110) YHYWTPEVTQ (120) GTPQTSGQQQ (130) QTWQKQAPTS (140) TMYQISPSQN (150) TSQQQTQSTQ (160) STMAGSWGGL (170) NQTQLRKTQA (180) YSSTTHMGWM (190) MATQPYNPNM (200) TTQNQTTYTT (210) QTTTAYQQQN (220) QNSSTTTLLL (230) SRTWNKLTWL (240) TPLIPSTQQS (250) QGGQPPQTGY (260) QPKWATTEEF (270) TKNNSQTTPT (280) TMATTTQQNQ (290) YYYQRLIYST (300) SITLLPMSNN (310) VKMKWQFEHT (320) KPTPFQPTQT (330) AQTTQQQPTS (340) PYMQMTL (347)

Seed: 1739065409

Google DeepMind About Google Google products Terms Output Terms Privacy Prohibited use policy Release Updates

p) NU3M^{QTY}

AlphaFold Server BETA   Server   About   FAQ & Guides

AlphaFold 3 code and weights are now available for local installation. Please see the FAQ for more information.   Dismiss

P03897_QTY

Back   Download   Clone and reuse   Feedback on structure

Very high (pIDDT > 90)   Confident (90 > pIDDT > 70)   Low (70 > pIDDT > 50)   Very low (pIDDT < 50)

ipTM = -   pTM = 0.6   learn more

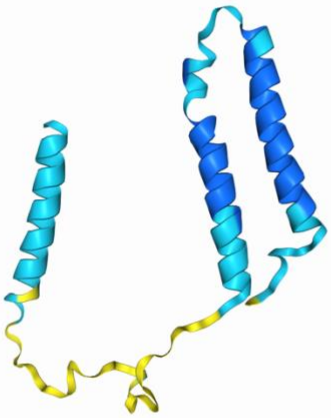

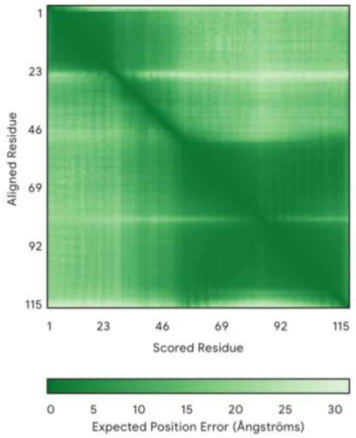

Non-commercial use only, subject to AlphaFold Server Output Terms of Use; no use in docking or screening tools.

Information

Type	Copies	Sequence
Protein	1	MNYAQTQMTN TQQAQQQMTT TYWLPQLNGY MEKSTPYECG FDPMSPARVP FSMKYYQTAT TYQQYDQETA QQQPQPWALQ TTNQPQMTMS SQQQTTTQAQ SQAYEWLQKG LDWTE

Seed: 250045434

Google DeepMind   About Google   Google products   Terms   Output Terms   Privacy   Prohibited use policy   Release Updates

q) NU4M^{QTY}

AlphaFold Server BETA   Server   About   FAQ & Guides

AlphaFold 3 code and weights are now available for local installation. Please see the FAQ for more information.   Dismiss

P03905_QTY

← Back   Download   Clone and reuse   Feedback on structure

Very high (pIDDT > 90)   Confident (90 > pIDDT > 70)   Low (70 > pIDDT > 50)   Very low (pIDDT < 50)

ipTM = -   pTM = 0.94   learn more

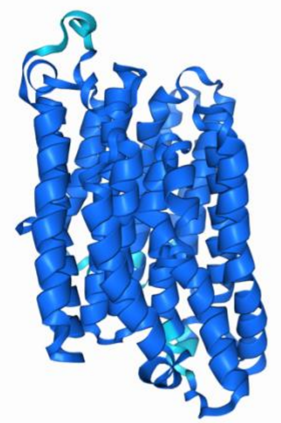

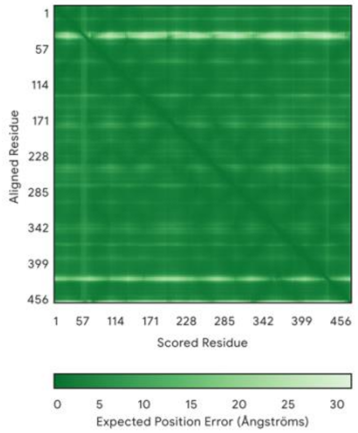

Non-commercial use only, subject to AlphaFold Server Output Terms of Use; no use in docking or screening tools.

Information

Type	Copies	Sequence
Protein	1	MLKLIVPTIM LLPLTWLSKK HMTWTNTTTH SQTTSTTPQQ YYNQINNNLF SCSPTFSSDP QTTPQQMQTT WQQPQTTMAS QRHLSSEPLS RKKLYLSMLI SQQTSQTMTY TATEQTMYYT YYETTLIPTL AIITRWGNQP ERLNAGTYYQ YYTQTGSQPQ QTAQTYTHNT LGSLNILLLT LTAQELSNSW ANNLMWQAYT MAYMTKMPQY GQHQWQPKAH VEAPTAGSMT QAATQQKQGG YGMMRLTLIL NPLTKHMAYP YQTQSQWGMT MTSSTCQRQT DLKSQTAYSS TSHMAQTTTA TQTQTPWSYT GATTQMTAHG QTSSQQYCQA NSNYERTHSR IMILSQGLQT QQPQMAYWWQ QASQANQAQP PTINLLGELS VLVTTFSWSN TTQQQTGQNM QTTAQYSQYM YTTTQWGSLT HHINNMKPSF TRENTLMFMH LSPILLLSLN PDIITGFSS

Seed: 530006216

Google DeepMind   About Google   Google products   Terms   Output Terms   Privacy   Prohibited use policy   Release Updates

r) NU5M^{QTY}

AlphaFold Server BETA Server About FAQ & Guides

AlphaFold 3 code and weights are now available for local installation. Please see the FAQ for more information. Dismiss

P03915_QTY

Back Download Clone and reuse Feedback on structure

Very high (pIDDT > 90) | Confident (90 > pIDDT > 70) | Low (70 > pIDDT > 50) | Very low (pIDDT < 50)

ipTM = - pTM = 0.92 learn more

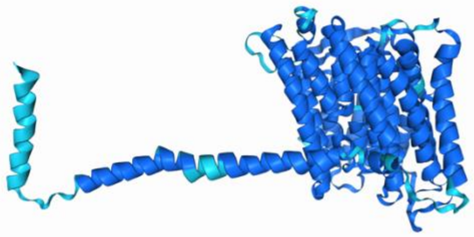

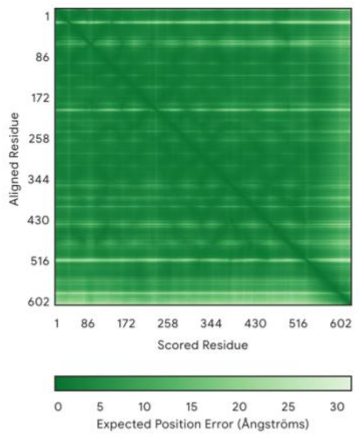

Non-commercial use only, subject to AlphaFold Server Output Terms of Use; no use in docking or screening tools.

Information

Type	Copies	Sequence
Protein	1	MTMHTTMTTL TLTSLIPPIL TTLVNPNKKN SYPHYVKSTT ASTYTTSQYP TTMYMCQDQE<br>VIISNWHWAT TQTTQLSLSF KLDYFSMMYT PTAQYTTWST MEYSQWYMNS DPNINQFFKY<br>LQTYQTTMQT QTTANNQYQQ YTGWEGTGTM SYQQTSWWYA RADANTAAIQ ATQYNRTGDT<br>GYTQAQAWYT QHSNSWDPQQ MALLNANPSL TPQQGQQQAA AGKSAQQGQH PWQPSAMEGP<br>TPTSAQQHSS TMTTAGTYQQ TRFHPLAENS PQTQTQTQCQ GATTTQYAAT CALTQNDIKK<br>TTAYSTSSQQ GQMMTTTGTN QPHLAYQHTC THAYYKAMQY MCSGSTTHNL NNEQDIRKMG<br>GLLKTMPLTS TSQTTGSQAQ AGMPYQTGYY SKDHIIETAN MSYTNAWAQS TTQTATSQTS<br>AYSTRMTQQT LTGQPRFPTL TNINENNPTL LNPIKRLAAG SQYAGYQTTN NTSPASPYQT<br>TTPQYQKQTA QATTYQGQQT AQDLNYLTNK LKMKSPLCTF YFSNMLGFYP SITHRTIPYL<br>GLLTSQNLPL LLLDLTWLEK LLPKTISQHQ ISTSIITSTQ KGMTKQYYQS YYYPQTQTQQ<br>QTT (603)

Seed: 176363115

Google DeepMind About Google Google products Terms Output Terms Privacy Prohibited use policy Release Updates

s) NU6M^{QTY}

AlphaFold Server BETA Server About FAQ & Guides

AlphaFold 3 code and weights are now available for local installation. Please see the FAQ for more information. Dismiss

P03923_QTY

← Back Download Clone and reuse Feedback on structure

Very high (pIDDT > 90) | Confident (90 > pIDDT > 70) | Low (70 > pIDDT > 50) | Very low (pIDDT < 50)

ipTM = - pTM = 0.7 learn more

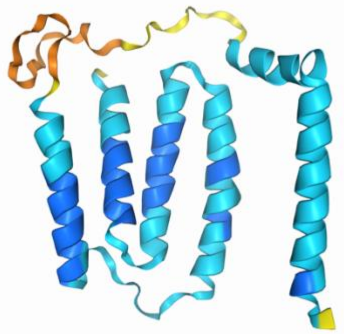

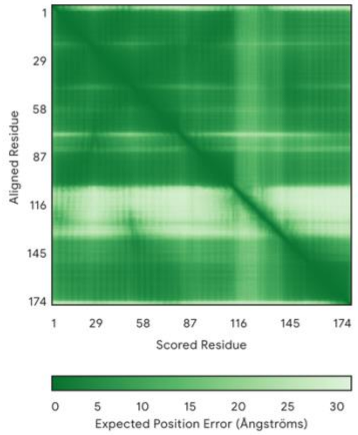

Non-commercial use only, subject to AlphaFold Server Output Terms of Use; no use in docking or screening tools.

Information

Type	Copies	Sequence
Protein	1	MMYAQYQQST GQTMGYTGYS SKPSPTYGGQ TQTTSGTTGC TTTQNYGGGY MGQMTYQTYQ GGMMTTYGYT TAMAIEEYPE AWGSGTETQT STQTGQAMET GQTQWTKEYD GTTTTTNYNS TGSWMTYEGE GSGLIREDPI GAGALYDYGR WQTTTTGWTQ YTGTYTTTET ARGN

Seed: 1014914494

Google DeepMind About Google Google products Terms Output Terms Privacy Prohibited use policy Release Updates

t) NU4LM^{QTY}

AlphaFold Server BETA Server About FAQ & Guides

P03901_QTY

Back Download Clone and reuse Feedback on structure

Very high (pIDDT > 90) | Confident (90 > pIDDT > 70) | Low (70 > pIDDT > 50) | Very low (pIDDT < 50)

ipTM = - pTM = 0.75 learn more

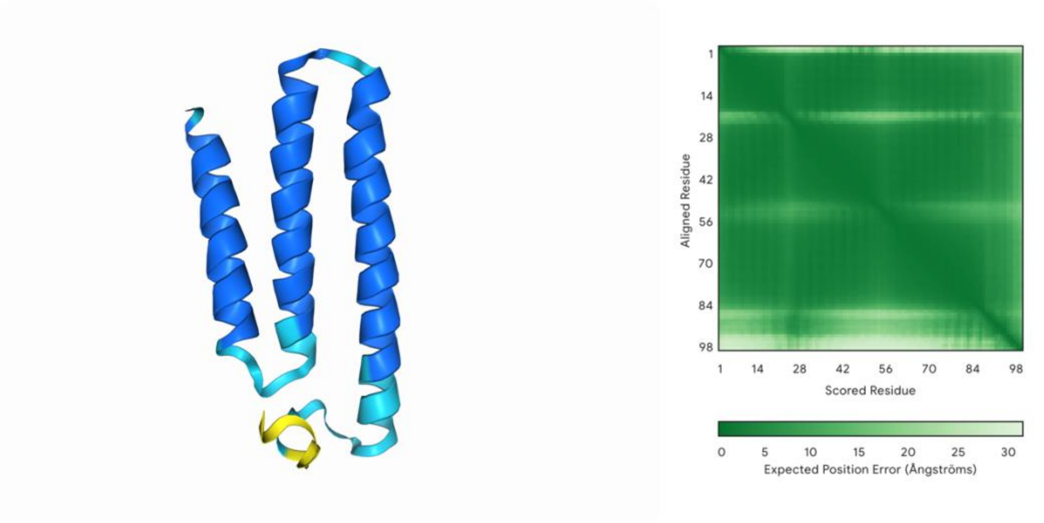

Non-commercial use only, subject to AlphaFold Server Output Terms of Use; no use in docking or screening tools.

Information

Type	Copies	Sequence
Protein	1	MPQTYMNTMQ AYTTSQQGMQ TYRSHLMSSQ QCQEGMMQSQ YTMATQMTQN THSLLANTTP TAMQTYAACE AATGQAQQVS ISNTYGLDYV HNLNLLQC

Seed: 1653259860

Google DeepMind About Google Google products Terms Output Terms Privacy Prohibited use policy Release Updates

u) Transmembrane arm of CI^{QTY}

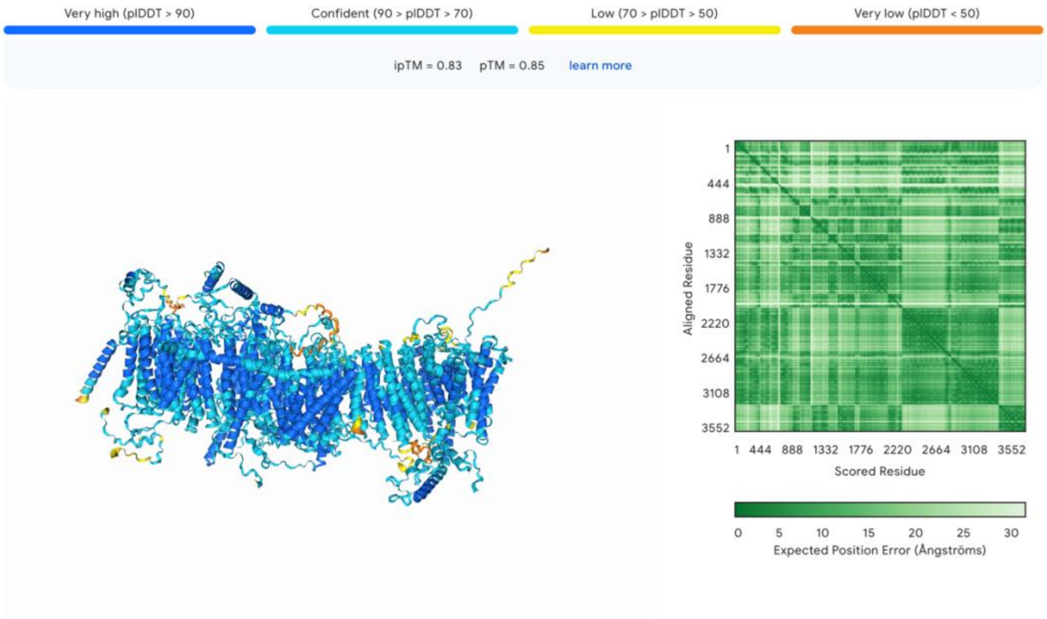

Supplement: Chen and Zhang supplementary material [file S263328922500002Xsup001.pdf]
